# Pb immobilization by phosphate-solubilizing fungi and fluorapatite under different Mn^2+^ concentrations

**DOI:** 10.3389/fmicb.2025.1696000

**Published:** 2025-10-29

**Authors:** Lei Zhang, Qiang Guan, Yifan Yan, Jiahui Sun, Linyue Xu, Shuo Zhang, Da Tian, Yue He

**Affiliations:** ^1^Ministry of Ecology and Environment Peoples Republic of China, Nanjing Institute of Environmental Science, Nanjing, China; ^2^Anhui Province Key Lab of Farmland Ecological Conservation and Pollution Prevention, Anhui Province Engineering and Technology Research Center of Intelligent Manufacture and Efficient Utilization of Green Phosphorus Fertilizer, College of Resources and Environment, Anhui Agricultural University, Hefei, China; ^3^Key Laboratory of Jianghuai Arable Land Resources Protection and Eco-restoration, Ministry of Natural Resources, College of Resources and Environment, Anhui Agricultural University, Hefei, China

**Keywords:** phosphate-solubilizing microorganisms, heavy metals, Pb remediation, organic acid, manganese

## Abstract

Phosphate-solubilizing fungi (PSF) are commonly employed in the bioremediation of lead contamination through the production of organic acids. However, the secretion of organic acids by PSF is typically affected by various environmental factors. This study investigated the Pb removal process by typical PSF *A. niger* and *P. chrysogenum* under different Mn^2+^ concentrations (0–30 mg/L). The different concentrations of Mn^2+^ can significantly influence the Pb toxicity tolerance of PSF. PSF *A. niger* exhibits a stable Pb removal ratio of >99% under different Mn^2+^ concentrations, much higher than *P. chrysogenum* <90%. The high concentrations of Mn^2+^ (15 and 30 mg/L) both inhibited the secretion of organic acids by *A. niger* and *P. chrysogenum*. However, 7.5 mg/L Mn^2+^ can significantly increase the secretion of oxalic acid by *A. niger* and promote the formation of lead oxalate and pyromorphite. Only 2.25% Pb^2+^ is released again from the immobilized Pb minerals. Meanwhile, PSF has the highest pyruvate dehydrogenase (PDH) enzyme activities of 31.53 and 17.23 nmol/min/g in 7.5 mg/L Mn^2+^ treatment. Compared with *P. chrysogenum*, *A. niger* is more effective in removing and stabilizing Pb cations. Controlling the appropriate Mn^2+^ concentration can further improve the Pb bioremediation by PSF.

## 1 Introduction

Lead (Pb) is widely recognized as a hazardous heavy metal, primarily due to the increased human activities such as mining, battery manufacturing, and paint utilization (Naik et al., 2013). The world’s most severe pollution issues are directly attributed to Pb (Raj and Das, 2023). In recent decades, more than 783,000 tons of Pb contaminants have been produced annually worldwide (Singh et al., 2003). As a persistent environmental contaminant, Pb in the environment has a long-term persistence and can cause high toxicity for organisms (Ozdemir et al., 2004; Naik et al., 2013; Zeng et al., 2017). Therefore, reducing the toxicity of Pb contamination in the environment is necessary and requires great attention in the future.

Bioremediation is an efficient pathway in reducing Pb contamination toxicity in the environment (Liang and Gadd, 2017). Compared with physical and chemical pathway, microbial remediation is more economical and environmentally friendly (Meng et al., 2024). Microorganisms can produce metabolites such as organic acids and extracellular polymeric substances (EPS) to immobilize Pb cantions (Priyadarshanee and Das, 2024). Additionally, the structure of cell waslls in microorganisms can also adsorb Pb cantions (Wang et al., 2019). For example, the fungal of *Aspergillus niger* (*A. niger*) can reduce more than 90% Pb cantions via the organic acid secretion and biosorption (Tian et al., 2019; Jalili et al., 2020). Meanwhile, the secretion of EPS from *A. niger* can also reduce Pb cantions concentration from 1,000 mg/L to 52.4 mg/L via the formation of EPS-Pb (Chen et al., 2018). Therefore, the microbial remediation of Pb exhibits considerable potential and merits further investigation.

Phosphate-solubilizing fungi (PSF) have been widely applied in Pb remediation. On the one hand, PSF exhibit a higher secretion of organic acids (e.g., oxalic acid, malic acid, citric acid, etc.) than other microorganisms (Coutinho et al., 2012; Sturm et al., 2015; Chowdhury et al., 2017). On the other hand, PSF typically has a a higher tolerance to Pb roxicity, which can maintaining organic acid secretion even at more than 1,000 mg/L Pb concentration (Huang et al., 2023). These organic acids can react or chelate with Pb cantions to form insoluble Pb minerals (e.g., lead oxalate), hence reducing the Pb concentration (Rhee et al., 2012). More importantly, these secreted organic acids can also dissolve insoluble phosphate (e.g., fluorapatite, FAp) and release phosphorus (P) (Echeverria et al., 2024). The released P can also react with Pb to form highly insoluble Pb minerals of pyromorphite (Pb_10_(PO_4_)_6_F_2_) (Park and Bolan, 2013). Hence, the PSF of *Aspergillus niger* is typically regarded as the leading candidate in Pb remediation, especially combined with FAp (Qiu et al., 2021).

Oxalic acid primarily facilitates the bioremediation of Pb by PSF via the formation of insoluble Pb mineral crystals, both around cell walls and in the external medium (Rhee et al., 2012; Osorio and Habte, 2013). Meanwhile, oxalic acid can also effectively promote the release of P from insoluble phosphate (Kpomblekou-A and Tabatabai, 2003). On the one hand, oxalic acid has the highest acidity constant (p*K*_a1_ = 1.25 and p*K*_a2_ = 4.27) compared with other organic acids such as citric acid (Palmieri et al., 2019). On the other hand, oxalic acid also has a higher chelating ability with metal cations (e.g., Pb^2+^, Ca^2+^, and Mg^2+^) due to its conjugated structure (Gadd, 1999). Therefore, promoting the secretion of oxalic acid is an effective strategy to enhance the Pb remediation by PSF.

Metal cation is essential to achieve the high secretion of organic acid by PSF (Li et al., 2017; Walaszczyk et al., 2018; Huang et al., 2023). However, the different metal cations concentration would affect the secretion of organic acid by PSF. Supply of divalent cations (e.g., Ca^2+^, Cu^2+^, etc.) can favor the secretion of organic acid by PSF, but influenced by the growth-limiting concentration (Palmieri et al., 2019). As a co-factor, the supply of Mn^2+^ can change the secretion of organic acid via the tricarboxylic acid (TCA) cycle, especially for oxalic acid (Ruijter et al., 1999). Oxalic acid is the primary pathway in Pb remediation by PSF. Therefore, the concentration of Mn^2+^ could also significantly affect the Pb remediation by PSF theoretically. However, there is insufficient research to confirm the importance and role of Mn^2+^ in Pb remediation by PSF.

This study aimed to investigate the effect of Mn^2+^ on Pb remediation by the PSF *Aspergillus niger* and *Penicillium chrysogenum* under the addition of fluorapatite (FAp). The capacity of P release from FAp between these two fungi was also investigated. The pH in the medium was measured by a pH meter. The oxalic acid concentration was measured by high-performance liquid chromatography (HPLC). The Pb and P concentrations in the solution were measured by inductively coupled plasma-optical emission spectrometry (ICP-OES). The resulting minerals were characterized by X-ray diffraction (XRD). The Pb in the mycelium was extracted by Toxicity Characteristic Leaching Procedure (TCLP).

## 2 Materials and methods

### 2.1 Fungal strains preparation

*Aspergillus niger* (*A. niger*) (CGMCC No. 23272) and *Penicillium chrysogenum* (*P. chrysogenum*) (CGMCC No. 23271) were isolated from the maize rhizosphere soil located in Suzhou, China (33^°^41′N, 117°5′E) (Wang et al., 2023; Feng et al., 2025). The Potato Dextrose Agar (PDA) medium was used to collect the spores for these two fungi. After 5 days of incubation at 28 °C, the formed fungal spores on the medium were drenched with sterile water using a fine artist’s brush. Subsequently, the mixture was filtered through a triple-layer sterile cheesecloth to remove mycelial fragments. Finally, a 0.85% sterile saline dilution was used and adjusted to 10^7^ cfu/mL using a hemocytometer.

### 2.2 Pb remediation by *A. niger* and *P. chrysogenum* under different Mn^2+^ conditions

The Pb(NO_3_)_2_ powder (Xilong Scientific Ltd.) was used as the Pb contamination in solution. The initial Pb concentration in the medium was 1,000 mg/L. MnCl_2_·4H_2_O (Macklin Inc.) was used as the source of Mn^2+^. For *A. niger* (ANG) and *P. chrysogenum* (PCH), five Mn^2+^ treatments were performed, i.e., 0 mg/L Mn^2+^, 3.75 mg/L Mn^2+^, 7.5 mg/L Mn^2+^, 15 mg/L Mn^2+^, and 30 mg/L Mn^2+^. Prior to the incubation process, 0.16 g of Pb(NO₃)₂ powder and 0.5 g of fluorapatite (FAp) were individually introduced into 150 mL Erlenmeyer flasks containing 100 mL of PDB medium. Then, 1 mL of *A. niger* and *P. chrysogenum* suspensions was added to each treatment, respectively. These flasks were incubated at 180 rpm and 28 °C under sterile conditions with the Parafilm (BS-QM-003, Biosharp) seals. After a 7-day incubation period, the PDB medium was collected and filtered through a 0.45 μm polyethersulfone (PES) membrane. The filtrates were collected for organic acids, pH, P concentration, and Pb content analysis. The centrifugal precipitates were collected to detect the enzyme activity. Meanwhile, the precipitates were also dried at 55 °C for 24 h to determine the dry biomass, XRD, and SEM analysis.

### 2.3 TPLC-Pb extracted from mycelium

The available Pb concentration leached from immobilized Pb minerals was tested by the TCLP method. The formed preceptaties with mycelium were mixed with the extraction solution (1:20) and shaken at 180 rpm for 18 ± 2 h at room temperature. The mixture was centrifuged, and the Pb concentration in the supernatant was measured by ICP-OES (Feng et al., 2025).

### 2.4 Enzyme activity assay

Using Pyruvate dehydrogenase (PDH) and citrate synthase (CS) activity assay kits (Comin Biotechnology Co., Ltd., Suzhou, China) to determine the enzyme activities from filtered fungal mycelium. Exhaustive methods refer to previous research (Tian et al., 2021).

### 2.5 Instrumentation

Using a pH meter (FE20, Mettler Toledo, Columbus, OH, United States) to determine the medium pH value. The concentration of P and Pb was analyzed by ICP-OES (PerkinElmer Avio 200, United States). Before the test, the filtrate was diluted 10 times. Calibration curves were prepared at concentrations of 1, 5, 10, 20, 50, and 100 mg/L using P and Pb standards (Feng et al., 2025).

The secretion of oxalic acid was determined by HPLC (Agilent 1200, Agilent Technologies, Santa Clara, CA, United States). The mobile phase included 2.5 wt‰ potassium dihydrogen phosphate (KH_2_PO_4_) and methanol (CH_3_OH) in a ratio of 99:1. The HPLC column temperature was 30 °C. Phosphoric acid was used to adjust the pH of KH_2_PO_4_ to 2.8 at a rate of 1 mL/min. Standard solutions of oxalic acid were diluted to concentrations of 1,500, 1,000, 500, 200, 100, 50, and 0 mg/L (Su et al., 2019).

The XRD analysis of precipitates was performed by D/Max-2500 X-ray diffraction (Rigaku Corporation, Tokyo, Japan, Cu-K; 36 kV; 20 mA; scanning from 5 to 60 at a speed of 4/s). Prior to XRD analysis, the filtered dry precipitates were ground in a planetary ball mill (Mitr YXQM, Changsha Mitrcn Instrument Equipment Co., Ltd., Changsha, China). The ground material was then passed through a 100-mesh sieve. Finally, using the MDI Jade 6.5 software to detect the results of XRD precipitation for phase identification.

### 2.6 Statistical analysis

Each experiment was replicated three times. The mean and standard deviation of each treatment were calculated and reported. Tukey’s honestly significant difference test (*p* < 0.05) was used to identify the significant differences among the treatments by one-way ANOVA. Before conducting one-way ANOVA, the data were tested for homogeneity of variance and normality using the Shapiro–Wilk and Levene tests. The data were analyzed statistically with SPSS 26.0 software.

## 3 Results

### 3.1 Fungal dry biomass and pH value in medium

**Figure 1:**
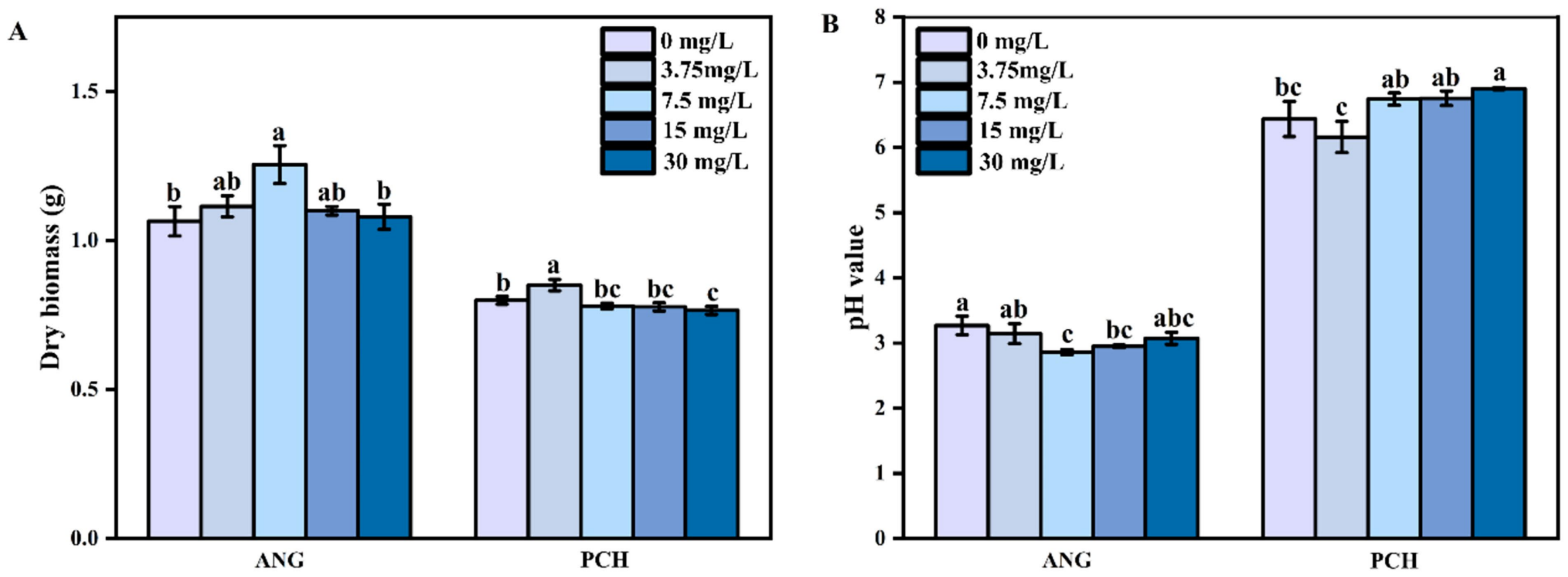
Dry biomass **(A)** and pH value **(B)** of *Aspergillus niger* and *Penicillium chrysogenum* in different Mn^2+^ conditions after 7 days of incubation. Standard deviations are shown with *N* = 3. The significant differences across treatments were carried out using Tukey’s honest significant difference test (*p* < 0.05) after one-way ANOVA. ANG, *Aspergillus niger*; PCH, *Penicillium chrysogenum*.

After 7 days of incubation, the fungal dry biomass in 0 and 3.75 mg/L Mn^2+^ treatments was 1.07 and 1.12 g (Figure 1A). In 7.5 mg/L Mn^2+^ treatment, the fungal dry biomass significantly increased to 1.26 g (Figure 1A). In 15 and 30 mg/L Mn^2+^ treatments, the fungal dry biomass decreased to 1.08 g and 1.10 g, respectively, (Figure 1A). For *P. chrysogenum*, the fungal dry biomass showed a lower value compared with *A. niger*, i.e., 0.80, 0.85, 0.78, 0.77, and 0.76 g in each Mn^2+^ concentration conditions after 7 days of incubation (Figure 1A).

The initial medium pH value is 6.5. After 7 days of incubation, the medium pH value in *A. niger* under 0, 3.75, 7.5, 15, and 30 mg/L Mn^2+^ treatments decreased to 3.27, 3.14, 2.86, 2.95, and 3.07, respectively (Figure 1B). For *P. chrysogenum*, the medium pH value showed a higher pH value compared to *A. niger*, i.e., ranged from 6.16 to 6.90 under different Mn^2+^ concentration, suggesting the lower secretion of organic acids from *P. chrysogenum* (Figure 1B).

### 3.2 Secretion of oxalic acid and citric acid by *A. niger* and *P. chrysogenum*

**Figure 2:**
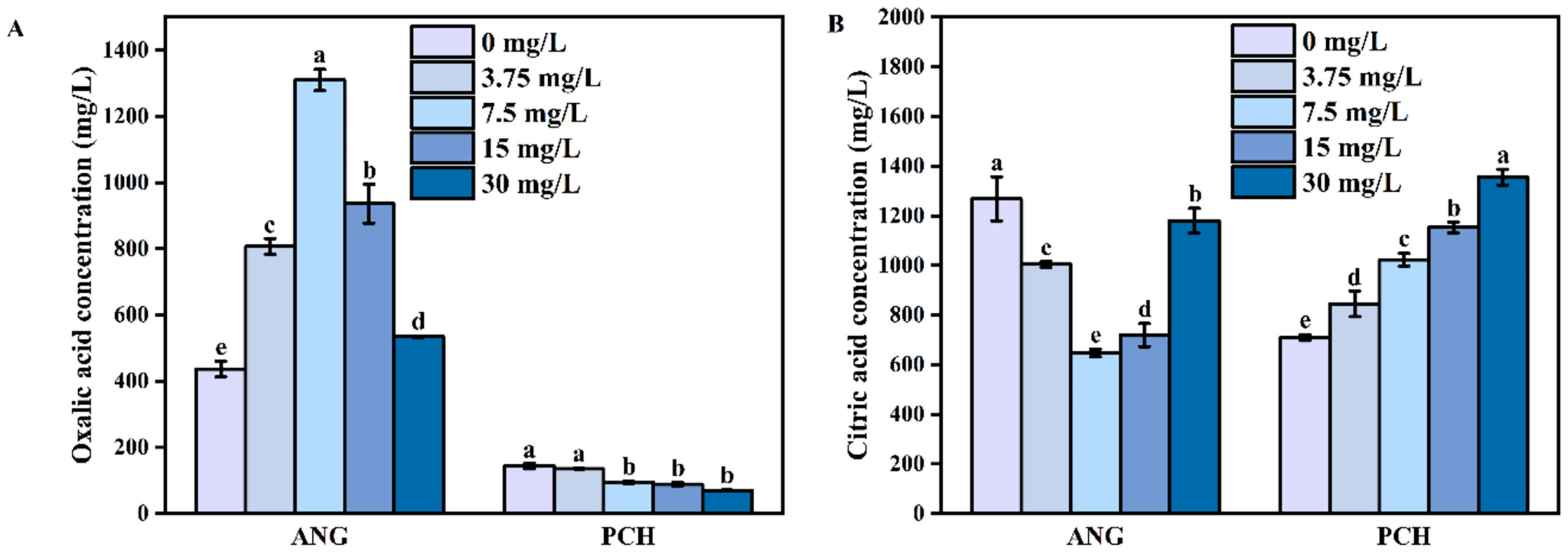
Oxalic acid **(A)** and citric acid content **(B)** *Aspergillus niger* and *Penicillium chrysogenum* in different Mn^2+^ conditions after 7 days of incubation. Standard deviations are shown with *N* = 3. The significant differences across treatments were carried out using Tukey’s honest significant difference test (*p* < 0.05) after one-way ANOVA. ANG, *Aspergillus niger*; PCH, *Penicillium chrysogenum*.

In 0 mg/L Mn^2+^ treatment, the concentration of oxalic acid secreted by *A. niger* was 436.87 mg/L (Figure 2A). Meanwhile, the oxalic acid concentration gradually increased to 807.01 mg/L in 3.75 mg/L Mn^2+^ treatment and reached to the highest value of 1310.16 mg/L in 7.5 mg/L Mn^2+^ treatment (Figure 2A). In 15 and 30 mg/L Mn^2+^ treatments, the secretion of oxalic acid remained the downward tendency (Figure 2A). For *P. chrysogenum*, the oxalic acid concentration was 144.12 mg/L in 0 mg/L Mn^2+^ treatment (Figure 2A). In 3.75, 7.5, 15, and 30 mg/L Mn^2+^ treatments, the oxalic acid secreted by *P. chrysogenum* was also shown a low value, ranging from 11.03 to 68.79 mg/L (Figure 2A).

Unlike oxalic acid, the citric acid secreted by *A. niger* in 7.5 mg/L Mn^2+^ treatment had the lowest value of 647.05 mg/L (Figure 2B). In 0 mg/L Mn^2+^ treatment, the citric acid concentration had the highest value of 1,268 mg/L (Figure 2B). Meanwhile, citric acid gradually decreased to 1004.40 mg/L in 3.75 mg/L Mn^2+^ treatment (Figure 2B). In addition, the concentrations of citric acid remained the upward tendency in 15 and 30 mg/L Mn^2+^ treatment (Figure 2B). For *P. chrysogenum*, the citric acid concentration was 709.52 mg/L without Mn^2+^ after 7 days of incubation. With the Mn^2+^ concentration increased to 3.75, 7.5, 15, and 30 mg/L, the secretion of citric acid by *P. chrysogenum* increased to 845.38, 1022.75, 1152.82, and 1355.10 mg/L, respectively (Figure 2B).

### 3.3 P concentration in the medium

**Figure 3:**
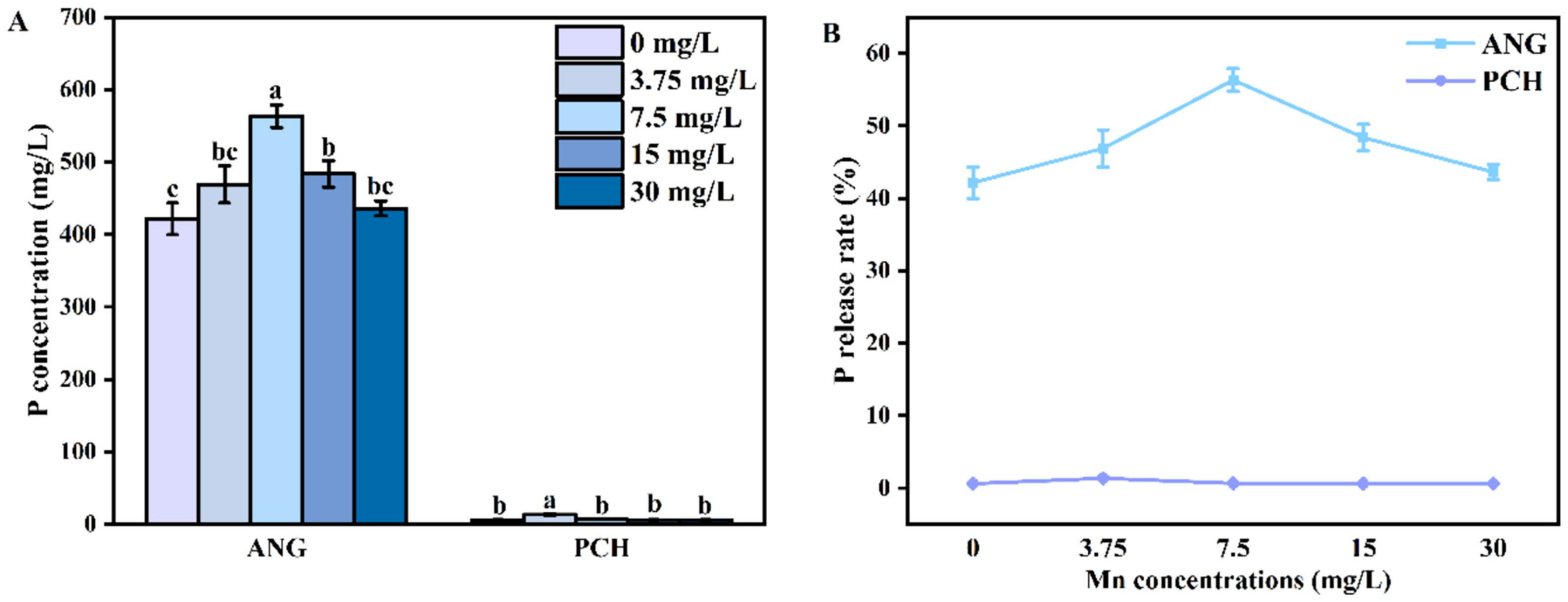
P concentration **(A)** and P release rate **(B)** of *Aspergillus niger* and *Penicillium chrysogenum* in different Mn^2+^ conditions after 7 days of incubation. Standard deviations are shown with *N* = 3. The significant differences across treatments were carried out using Tukey’s honest significant difference test (*p* < 0.05) after one-way ANOVA. ANG, *Aspergillus niger*; PCH, *Penicillium chrysogenum*.

The P concentration in *A. niger* medium without Mn^2+^ addition (0 mg/L) was 421.53 mg/L (Figure 3A). In 3.75 and 7.5 mg/L Mn^2+^ treatments, the content of P increased to 469.95 and 563.27 mg/L, respectively (Figure 3A). In 15 and 30 mg/L Mn^2+^ treatments, the P concentration decreased to 483.79 and 436.14 mg/L, respectively (Figure 3A). For *P. chrysogenum*, the P was hardly released from FAp. The highest P content was 13.38 mg/L in 3.75 mg/ L Mn^2+^ treatment (Figure 3A). In other Mn^2+^ treatments, the P content ranged from 6.08 to 6.56 mg/L (Figure 3A). Compared with *P. chrysogenum*, *A. niger* is more efficient in the release of P from FAp, i.e., more than 40% P release rate by *A. niger* vs. less than 5% P release rate by *P. chrysogenum* (Figure 3B).

### 3.4 Pb remediation by *A. niger* and *P. chrysogenum* under different Mn^2+^ conditions

**Figure 4:**
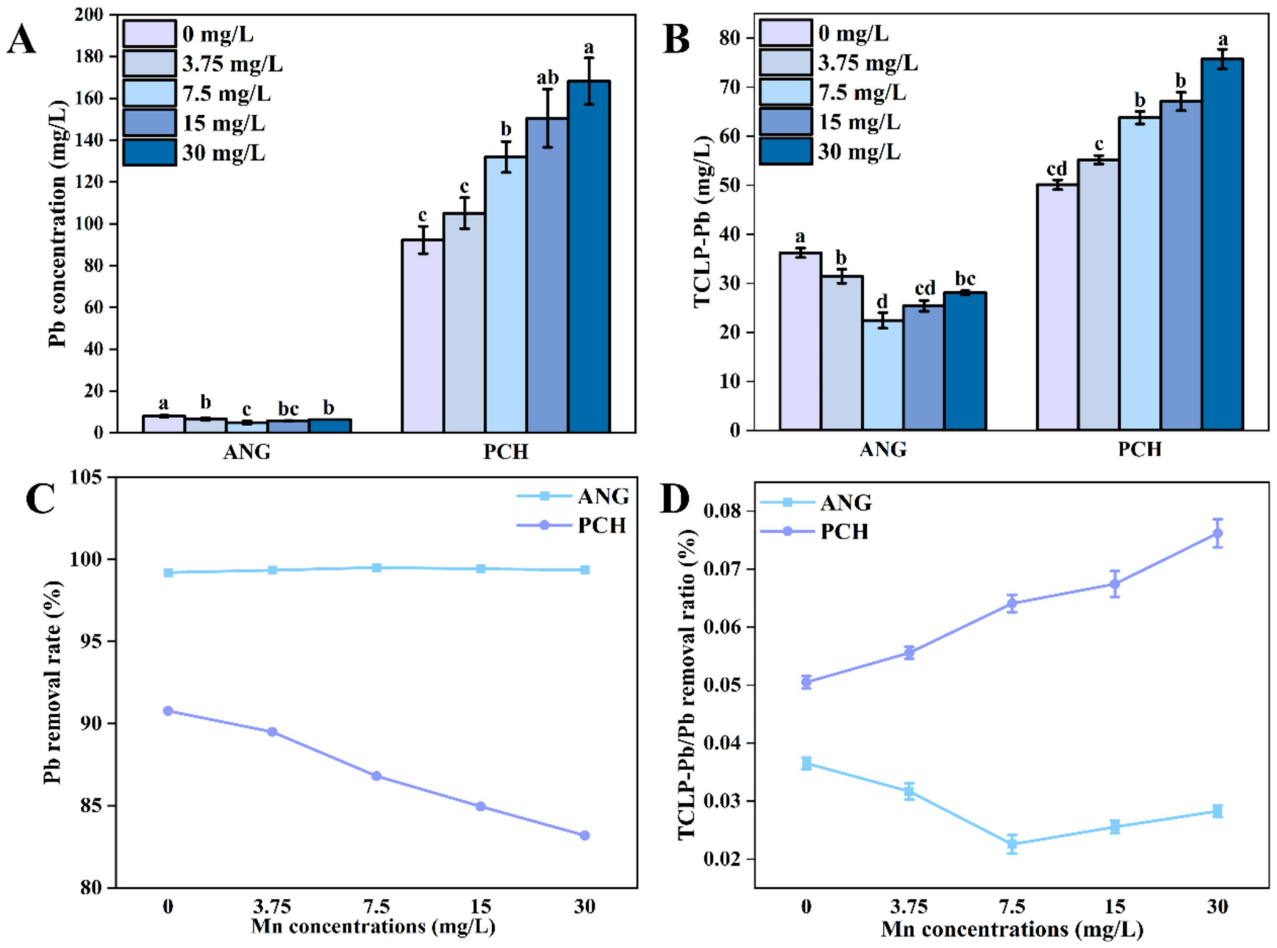
Pb^2+^ content in solution **(A)**, TCLP-Pb concentration **(B)**, Pb removal ratio **(C)**, and TCLP-Pb/removed Pb ratio **(D)** of *Aspergillus niger* and *Penicillium chrysogenum* in different Mn^2+^ conditions after 7 days of incubation. Standard deviations are shown with *N* = 3. The significant differences across treatments were carried out using Tukey’s honest significant difference test (*p* < 0.05) after one-way ANOVA. ANG, *Aspergillus niger*; PCH, *Penicillium chrysogenum*.

After 7 days of incubation, the Pb concentration in each Mn^2+^ treatments was significantly decreased from 1,000 mg/L to 8.00, 6.59, 4.94, 5.80, and 6.40 mg/L in *A. niger*, respectively (Figure 4A). However, the Pb contents in different Mn^2+^ treatments with *P. chrysogenum* had about 10 times than *A. niger* (Figure 4A). The Pb concentration in *P. chrysogenum* under different Mn^2+^ treatment ranged from 93.31 to 168.17 mg/L after 7 days of incubation (Figure 4A).

The TCLP-Pb concentration in *A. niger* with 0, 3.75, 7.5, 15, 30 mg/L Mn^2+^ treatments were 36.23, 31.47, 22.43, 25.39, and 28.09 mg/L after 7 days of incubation, respectively (Figure 4B). For *P. chrysogenum*, the TCLP-Pb concentration under different Mn^2+^ treatments showed a higher value of 50.10, 55.20, 63.77, 67.06, and 75.71 mg/L (Figure 4B). Meanwhile, the Pb removal ratio in *A. niger* was much higher than *P. chrysogenum* under different Mn^2+^ treatments, i.e., 99.1 to 99.5% vs. 83.1 to 90.7% (Figure 4C). In addition, the ratio of TCLP-Pb/immobilized Pb between *A. niger* and *P. chrysogenum* in 0, 3.75, 7.5, 15, 30 mg/L Mn^2+^ treatments was 3.65, 3.17, 2.25, 2.55, 2.83 and 5.27%, 5.84, 6.81, 7.19, 8.19%, respectively (Figure 4D).

### 3.5 Enzyme activity in *A. niger* and *P. chrysogenum* under different Mn^2+^ conditions

**Figure 5:**
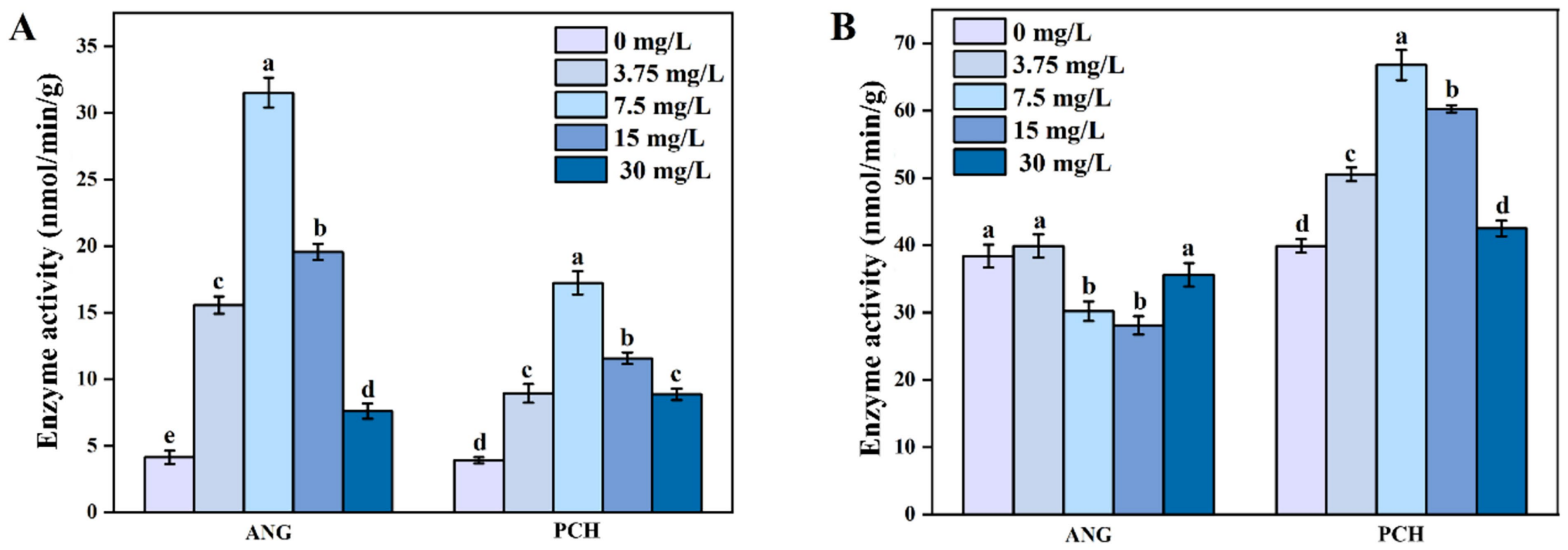
Pyruvate dehydrogenase (PDH) enzyme activities **(A)** and citrate synthase (CS) enzyme activities **(B)** of *Aspergillus niger* and *Penicillium chrysogenum* in different Mn^2+^ conditions after 7 days of incubation. Standard deviations are shown with *N* = 3. The significant differences across treatments were carried out using Tukey’s honest significant difference test (*p* < 0.05) after one-way ANOVA. ANG, *Aspergillus niger*; PCH, *Penicillium chrysogenum*.

The enzyme activity of pyruvate dehydrogenase (PDH) in both *A. niger and P. chrysogenum* initially increased and then decreased as the Mn^2+^ concentration increased from 0 to 30 mg/L (Figure 5A). In 7.5 mg/L Mn^2+^ treatment, *A. niger and P. chrysogenum* showed the highest enzyme activity, i.e., 31.53 and 17.23 nmol/min/g, respectively (Figure 5A). In 0, 3.75, 15, and 30 mg/L Mn^2+^ treatments, the PDH activity in *A. niger and P. chrysogenum* ranged from 4.13 ~ 19.57 and 3.90 ~ 11.57 nmol/min/g, respectively (Figure 5A). For citrate synthase (CS) enzyme activity, *P. chrysogenum* showed a similar trend with PDH, i.e., reached the highest value of 66.77 nmol/min/g in 7.5 mg/L Mn^2+^ treatment and ranged from 39.90 to 60.27 nmol/min/g (Figure 5B). The CS enzyme activity in *A. niger* under 0, 3.75, 15, and 30 mg/L Mn^2+^ treatments were 38.40, 39.90, 30.20, 28.10, and 35.60 mg/L, respectively (Figure 5B).

### 3.6 XRD analysis and mineral peak area ratio

**Figure 6:**
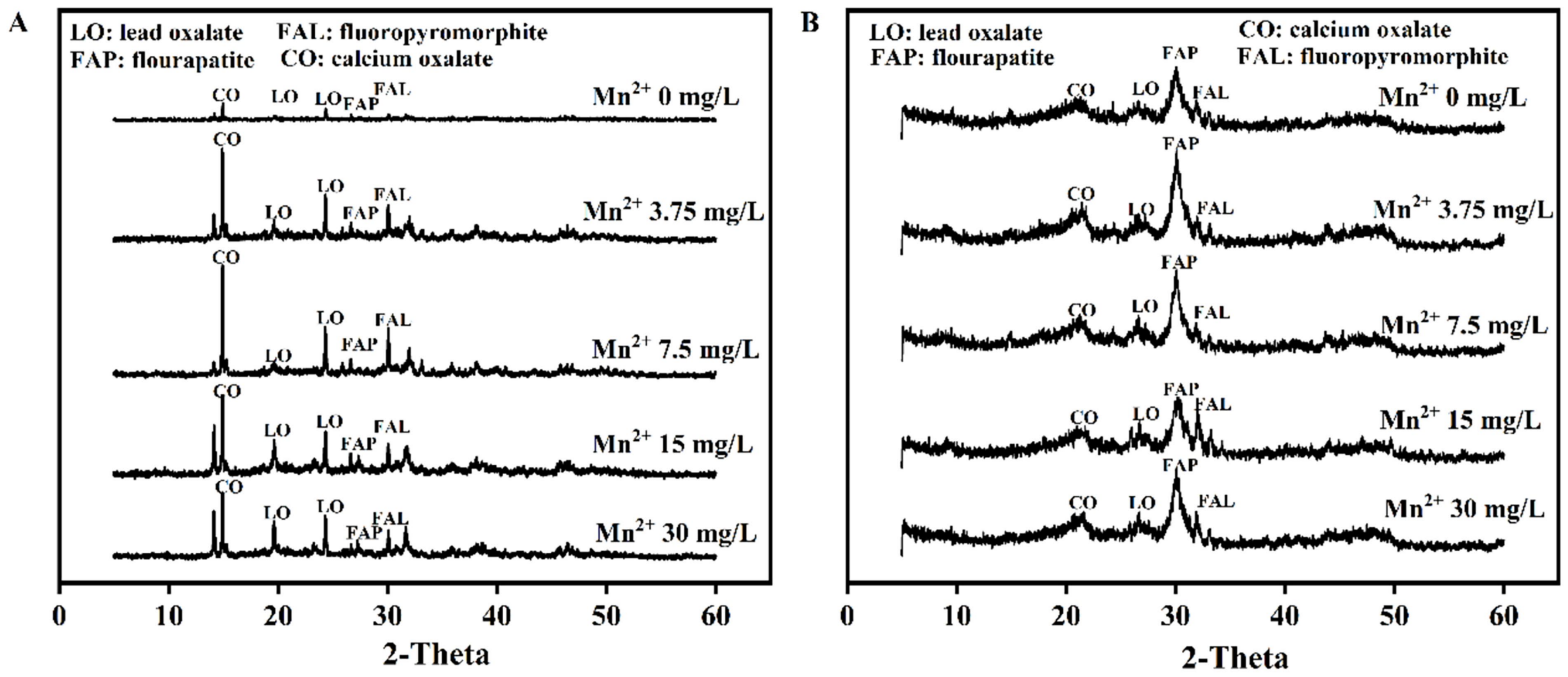
XRD patterns of *Aspergillus niger* **(A)** and *Penicillium chrysogenum* **(B)** under different Mn^2+^ concentrations after 7 days of incubation.

**Figure 7:**
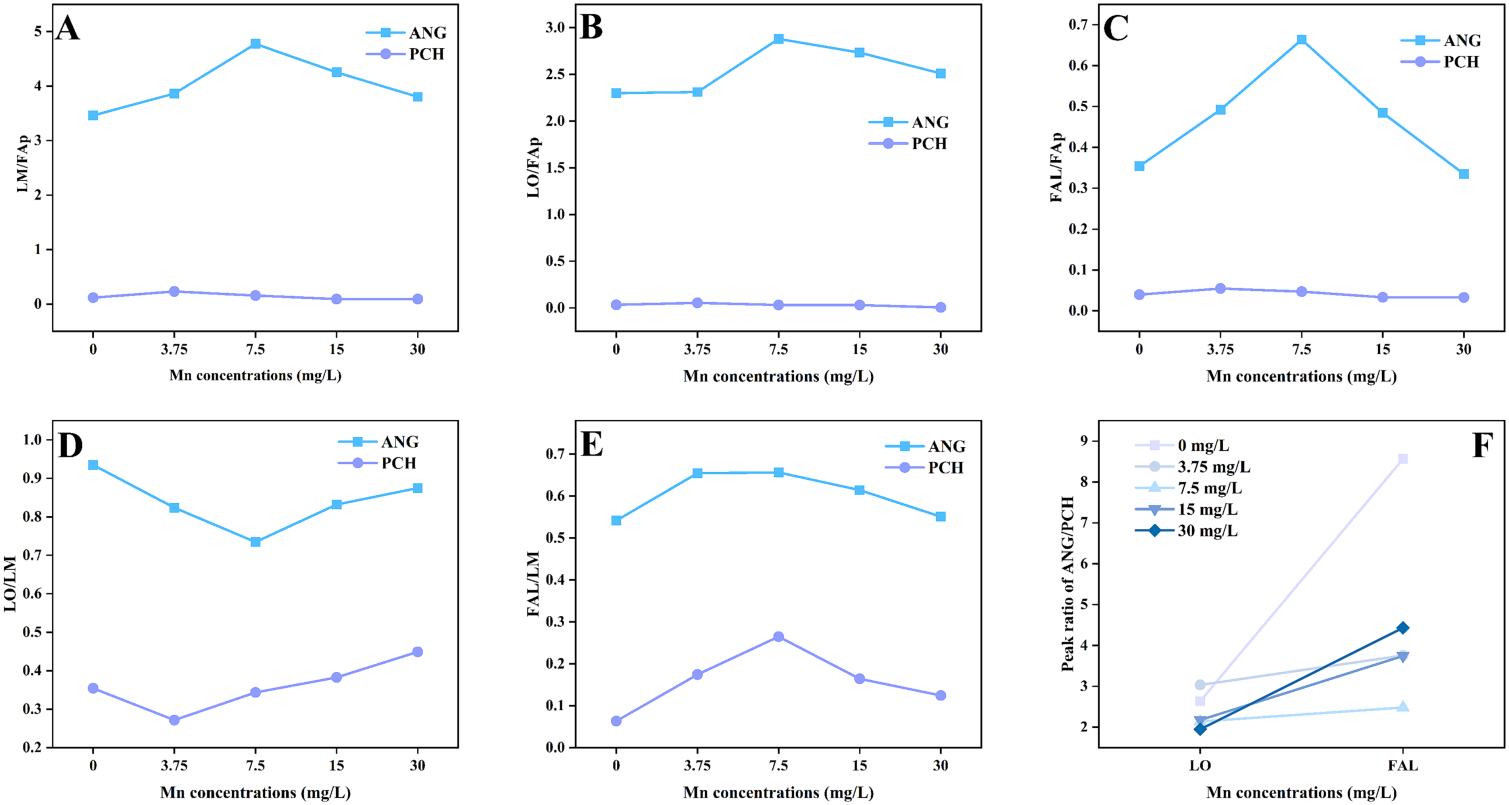
Peak area ratio of Pb minerals of *Aspergillus niger* and *Penicillium chrysogenum* in different Mn^2+^ conditions after 7 days of incubation. **(A–E)** Peak area ratio of LM/FAp, LO/FAp, FAL/FAp, LO/LM, and FAL/LM between ANG and PCH. **(F)** The peak area ratio between ANG and PCH in the formed LO and FAL. LO, lead oxalate; FAp, fluorapatite; FAL, fluoropyromorphite; LM, lead minerals (LO + FAL); ANG, *Aspergillus niger*; PCH, *Penicillium chrysogenum*.

XRD patterns showed the Pb mineralization by *A. niger* and *P. chrysogenum* at Mn^2+^ concentrations of 0, 3.75, 7.5, 15, and 30 mg/L (Figure 6). The peaks located at 24.32° and 31.74° represent the minerals of lead oxalate (LO) and fluoropyromorphite (FAL) (Chen et al., 2019). In *A. niger* treatment, the peaks of LO and FAL were clearly observed at 3.75, 7.5, 15, and 30 mg/L Mn^2+^ concentrations (Figure 6A). For *P. chrysogenum*, the peaks of LO and FAL were also observed (Figure 6B). The peak area ratios of minerals between *A. niger* and *P. chrysogenum* are shown in Figure 7. For *A. niger*, the area ratio of LM (LO + FAL)/FAp was much higher than *P. chrysogenum*, i.e., 3.46 ~ 4.77 vs. 0.09 ~ 0.23 (Figure 7A). Under different Mn^2+^ treatments, the peak area ratios of LO/FAp were 2.30, 2.31, 2.88, 2.73, and 2.51 in *A. niger*, much higher than 0.033, 0.054, 0.031, 0.030, and 0.005 in *P. chrysogenum* (Figure 7B). Meanwhile, the peak area ratios of FAL/FAp in *A. niger* also higher than *P. chrysogenum*, i.e., 0.35, 0.49, 0.66, 0.48, and 0.33 vs. 0.04, 0.0550, 0.046, 0.033, and 0.032 (Figure 7C). The peak area ratios of LO/LM were 0.93, 0.82, 0.73, 0.83, and 0.87 in *A. niger*, while in *P. chrysogenum* was only 0.35, 0.27, 0.34, 0.38, and 0.44 (Figure 7D). The peak area ratios of FAL/LM were 0.54, 0.65, 0.66, 0.61, 0.55 in *A. niger* and 0.06, 0.17, 0.26, 0.16, 0.12 in *P. chrysogenum* (Figure 7E). The XRD peak area ratio of LO and FAL formed in *A. niger* were 2.64, 3.03, 2.14, 2.18, 1.95 and 8.56, 3.76, 2.48, 3.74, 4.43 times than *P. chrysogenum*, respectively (Figure 7F).

### 3.7 SEM analysis

**Figure 8:**
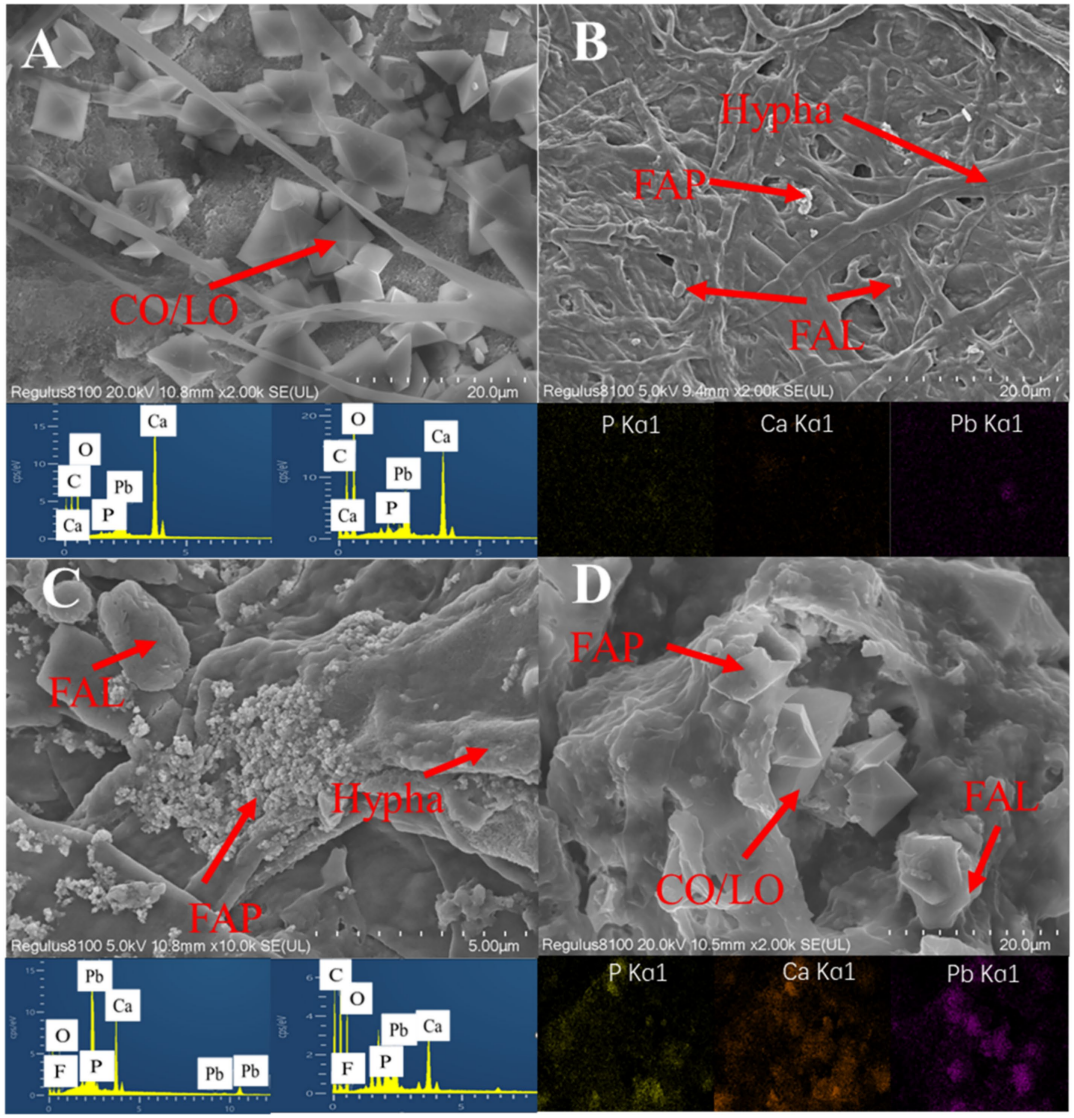
SEM and EDS mapping images of *Aspergillus niger* and *Penicillium chrysogenum* after 7 days of incubation. **(A,B)** *A. niger* + Pb + FAp. **(C,D)** *P. chrysogenum* + Pb + FAp. LO, lead oxalate; CO, calcium oxalate; FAp, fluorapatite; FAL, fluoropyromorphite.

SEM images of *Aspergillus niger* (7.5 mg/L Mn^2+^) and *Penicillium chrysogenum* (3.75 mg/L Mn^2+^) after 7 days of incubation were shown in Figure 8. *Aspergillus niger* exhibited robust growth after 7 days, characterized by significant mycelial development encapsulated with calcium oxalate, lead oxalate, and fluoropyromorphite (Figures 8A,B). Similarly, in the presence of *Penicillium chrysogenum*, the culture displayed the presence of calcium oxalate, lead oxalate, and fluoropyromorphite around the ruptured *Penicillium chrysogenum* (Figures 8C,D). These findings indicated that the ability of both *Aspergillus niger* and *Penicillium chrysogenum* can dissolve FAp and promote Pb immobilization.

## 4 Discussion

Bioremediation is a high-efficiency and low-cost pathway for Pb remediation (Dang et al., 2018; Feng et al., 2022; Guan et al., 2024). This research shows that both *A. niger* and *P. chrysogenum* can tolerate a high Pb toxicity (1,000 mg/L) and remove more than 83% Pb^2+^ in the solution (Figure 4). Especially for *A. niger*, the combination of FAp removed more than 99.3% Pb^2+^, not only higher than *P. chrysogenum*, but also more efficient than other fungi, e.g., red yeast combine calcium phosphate remove 90.64% Pb^2+^ (Feng et al., 2022; Tian et al., 2022). Meanwhile, the Pb immobilized by *A. niger* is more stable than *P. chrysogenum*. In *A. niger*, less than 3.7% Pb^2+^ can be re-released from the immobilized Pb minerals, much lower than the 5.3–8.2% observed in *P. chrysogenum* (Figure 4). Therefore, *A. niger* is more efficient in Pb remediation compared with *P. chrysogenum*.

Organic acid plays a key role in Pb remediation by PSF and FAp, determined the efficiency of Pb immobilization (Li et al., 2016; Meng et al., 2022). Our research indicates that the secretion of oxalic acid in *A. niger* is 5–10 times higher than in *P. chrysogenum* (Figure 2). Previous research confirmed that *A. niger* has a stronger ability to secrete oxalic acid compared to other PSF, such as *Penicillium oxalicum* (Tian et al., 2019; Tian et al., 2024). Therefore, oxalic acid would not be the primary mechanism for *P. chrysogenum* in Pb immobilization. In addition, the dry biomass of *P. chrysogenum* was also much lower than *A. niger*, i.e., 0.76–0.85 g vs. 1.07–1.26 g (Figure 1B). In other words, a 1,000 mg/L Pb concentration inhibits the growth of *P. chrysogenum* and its secretion of oxalic acid. Hence, the other process like bioadsorption may dominated the Pb immobilization by *P. chrysogenum*. Compared with other organic acid (e.g., citric acid), oxalic acid has a higher acidity constants (p*K*a = 1.25) and chelating capacity than other organic acid (Li et al., 2016; Palmieri et al., 2019). Thus, *A. niger* has a higher Pb removal ratio than *P. chrysogenum*, i.e., 99% vs. ~90% (Figure 4). Previous research has demonstrated that the secretion of oxalic acid by PSF primarily drives the dissolution of phosphate and the remediation of Pb (Jiang et al., 2020). Especially for PSF, enhancing the immobilization capacity of Pb can be achieved by promoting the secretion of oxalic acid (Tian et al., 2023).

The oxalic acid secretion by PSF would also influence the stability of the removed Pb minerals (Wang et al., 2016; Feng et al., 2025). In Pb bioremediation, the stability of immobilized Pb is usually depended on the formed Pb minerals (Feng et al., 2025). In this research, both XRD patterns and SEM images confirmed the formation of LO and FAL in both *A. niger* and *P. chrysogenum* (Figures 6, 8). However, *A. niger* shows a higher stability of immobilized Pb compared to *P. chrysogenum* (Figure 4). Due to the high secretion of oxalic acid, *A. niger* is more effective in forming Pb minerals when combined with FAp (Mendes et al., 2020). The secreted oxalic acid not only forms insoluble LO by chelating with Pb (Equation 1) but also facilitates the release of P from phosphate and reacts with Pb to create highly insoluble fluoropyromorphite (Equations 2, 3) (Ma and Rao, 1999; Tian et al., 2023). The formed Pb minerals of LO and FAL by PSF demonstrated the Pb immobilization and contributed to its stability (Giammar et al., 2008; Ahemad, 2015; Feng et al., 2025).


(1) $${\mathrm{Pb}}^{2+}+C_{2}O_{4}^{2–}\Leftrightarrow {\mathrm{PbC}}_{2}O_{4}$$

(2) $${\mathrm{Ca}}_{5}{\left({\mathrm{PO}}_{4}\right)}_{3}F+{\mathrm{6H}}^{+}\Leftrightarrow {\mathrm{5Ca}}^{2+}+{\mathrm{3H}}_{2}{\mathrm{PO}}_{4}^{-}+F^{-}$$

(3) $${\mathrm{5Pb}}^{2+}+{\mathrm{3H}}_{2}{\mathrm{PO}}_{4}^{-}+F^{-}\Leftrightarrow {\mathrm{Pb}}_{5}{\left({\mathrm{PO}}_{4}\right)}_{3}F+{\mathrm{6H}}^{+}$$

Meanwhile, the XRD peak area ratio shows that the Pb minerals of LO and FAL formed in *A. niger* are ~2 and ~4 times than *P. chrysogenum*, respectively (Figure 7). LO and FAL are the primary pathways in Pb immobilization using the combination of PSF and FAp, which have a low solubility (Li et al., 2016; Tian et al., 2019). Therefore, the different oxalic acid secretion significantly changed the stability of the removed Pb between *A. niger* and *P. chrysogenum* (Figure 4).

Metal cations can significantly influence the the secretion of organic acids by fungi (Behera, 2020). The addition of metal cations (Ca^2+^, Mg^2+^, and Mn^2+^, etc.) not only affects the growth of PSF but also influences the secretion of oxalic acid (Kobayashi et al., 2014; Geng et al., 2022). In this research, the addition of Mn^2+^ significantly changed the secretion of organic acid between *A. niger* and *P. chrysogenum* (Figure 2). For *A. niger*, low Mn^2+^ concentrations (0 and 3.75 mg/L) and high Mn^2+^ concentrations (15 and 30 mg/L) both limited oxalic acid secretion but enhanced citric acid secretion (Figure 2). In contrast, the high Mn^2+^ concentrations stimulated the secretion of citric acid in *P. chrysogenum* (Figure 2). These differences in organic acid secretion can also alter the Pb immobilization capacity of PSF (Tian et al., 2019). Our research indicates that the addition of Mn^2+^ enhances the secretion of oxalic acid by *A. niger* and citric acid by *P*. *chrysogenum,* respectively. Compared to *P. chrysogenum*, *A. niger* is more effective in Pb remediation across various Mn^2+^ concentrations.

Mn^2+^ has been demonstrated to be the co-factor that alters the secretion of organic acids by fungi (Arvieu et al., 2003). Moderate amounts of Mn^2+^ can promote the growth of fungi and improve their resistance to different environmental stresses (Berovic et al., 2006; Fekete et al., 2022). In contrast, the deficiency or excess Mn^2+^ would also inhibit fungal growth and alter metabolic pathways (Pinzari et al., 2018). In *A. niger* and *P. chrysogenum*, the fungal growth and organic acid secretion are different under different Mn^2+^ concentrations. This difference could be attributed to the physiological traits and metabolic pathways of the two fungi (Huang et al., 2013). In addition, differences in metabolic pathways and gene expression between these two fungi further influence their response and adaptation mechanisms to Mn^2+^ (Carrasco et al., 2011; Xie et al., 2018). *A. niger* has strong environmental adaptability and stress resistance and can survive and reproduce in a variety of environmental conditions (Qiu et al., 2021). *P. chrysogenum* is sensitive to environmental conditions and vulnerable to external factors, e.g., pH value, phosphate source, etc. (Bodini et al., 2011; Wang et al., 2023). Our previous research has confirmed that the pH > 5.5 condition could decrease the stability of Pb immobilization (Feng et al., 2025). Notably, all of the pH value in *P. chrysogenum* under different Mn^2+^ concentrations exceeded 6 (pH > 6), which might explain the lower Pb remove ratio and stability of immobilized Pb.

The tricarboxylic acid (TCA) cycle is the main pathway for organic acid secretion in PSF, determining both the quantity and types of secreted acids (Mäkelä et al., 2010; Beatrice et al., 2022). First, pyruvate dehydrogenase (PDH) catalyzes the conversion of pyruvate to acetyl-CoA, thereby facilitating the production of organic acids in the TCA cycle (Mattevi et al., 1992; Dutton and Evans, 1996). Meanwhile, citrate synthase (CS) catalyzes the synthesis of citric acid in PSF (Alekseev et al., 2017; Behera, 2020). The enzyme activity regulated the secretion of organic acids, as well as the phosphate solubilization and Pb remediation facilitated by PSF (Schmalenberger et al., 2015; Feng et al., 2022). Our research also indicates that the PDH activity in *A. niger* is higher than *P. chrysogenum* (Figure 5). Meanwhile, the activity of CS enzyme in *P. chrysogenum* is much higher than in *A. niger*, which aligns with the higher secretion of citric acid (Figure 5). In addition, metal cations can influence the organic acid secretion by affecting the enzyme activity. Fe^3+^ can regulate CS enzyme activity and enhance the secretion of citric acid by PSF (Hao et al., 2021; Wang et al., 2023). Similarly, the 7.5 mg/L Mn^2+^ can significantly increase the PDH enzyme activity in *A. niger* and *P. chrysogenum* (Figure 5A). Meanwhile, the 7.5 mg/L Mn^2+^ can also significantly increase the CS enzyme activity in *P. chrysogenum* (Figure 5A). Therefore, controlling the Mn^2+^ concentration is important for regulating the metabolism of organic acid and Pb remediation in PSF.

## 5 Conclusion

The combination of PSF *A. niger* and *P. chrysogenum* with FAp can effectively remove Pb cations via the formation of stable Pb minerals. Oxalic acid secreted by *A. niger* and *P. chrysogenum* plays a dominate role in Pb removal. Notably, *A. niger* exhibits a higher oxalic acid secretion compared to *P. chrysogenum*, which enhances the formation of insoluble lead oxalate and pyromorphite. An optimal Mn^2+^ concentration (7.5 mg/L) significantly stimulates oxalic acid secretion by *A. niger*, hence accelerating FAp dissolution and promoting pyromorphite formation. However, suboptimal (≤7.5 mg/L) or excessive (15–30 mg/L) Mn^2+^ concentrations reduce the stability of Pb immobilization by *A. niger*. In summary, *A. niger* is more effective than *P. chrysogenum* in Pb remediation, and an appropriately regulated Mn^2+^ concentration further enhances its Pb removal capacity.

## Data Availability

The original contributions presented in the study are included in the article/supplementary material, further inquiries can be directed to the corresponding authors.
